# Competition between movement plans increases motor variability: evidence of a shared resource for movement planning

**DOI:** 10.1152/jn.00113.2016

**Published:** 2016-06-29

**Authors:** Leonie Oostwoud Wijdenes, Richard B. Ivry, Paul M. Bays

**Affiliations:** ^1^Institute of Neurology, University College London, London, United Kingdom;; ^2^Donders Institute for Brain, Cognition and Behaviour, Radboud University Nijmegen, Nijmegen, The Netherlands;; ^3^Institute of Cognitive and Brain Sciences, University of California, Berkeley, California; and; ^4^Department of Psychology, University of Cambridge, Cambridge, United Kingdom

**Keywords:** movement planning, motor control, parallel encoding, action, reaching

## Abstract

Do movement plans, like representations in working memory, share a limited pool of resources? If so, the precision with which each individual movement plan is specified should decrease as the total number of movement plans increases. To explore this, human participants made speeded reaching movements toward visual targets. We examined if preparing one movement resulted in less variability than preparing two movements. The number of planned movements was manipulated in a delayed response cueing procedure that limited planning to a single target (*experiment 1*) or hand (*experiment 2*) or required planning of movements toward two targets (or with two hands). For both experiments, initial movement direction variability was higher in the two-plan condition than in the one-plan condition, demonstrating a cost associated with planning multiple movements, consistent with the limited resource hypothesis. In *experiment 3*, we showed that the advantage in initial variability of preparing a single movement was present only when the trajectory could be fully specified. This indicates that the difference in variability between one and two plans reflects the specification of full motor plans, not a general preparedness to move. The precision cost related to concurrent plans represents a novel constraint on motor preparation, indicating that multiple movements cannot be planned independently, even if they involve different limbs.

## Abstract (precis)

*Various lines of evidence indicate that multiple movements can be prepared in parallel. Here, we show that preparing more than one movement comes with a cost: a movement plan is more variable if it is prepared simultaneously with another plan. This suggests that the representations of movement plans share a common neural resource and implies that the number of alternative plans is constrained by noise*.

## NEW & NOTEWORTHY

*Various lines of evidence indicate that multiple movements can be prepared in parallel. Here, we show that preparing more than one movement comes with a cost: a movement plan is more variable if it is prepared simultaneously with another plan. This suggests that the representations of movement plans share a common neural resource and implies that the number of alternative plans is constrained by noise*.

a simple decision, such as selecting which of two identical cartons of milk to grab from the supermarket shelf, involves both perceptual processes, to determine the locations of the cartons, and motor processes, to prepare the appropriate movement (Song and Nakayama 2009). Neurophysiology studies with nonhuman primates have indicated that choosing between two possible actions is not a serial process, whereby one of the two actions is selected and then the selected action is planned and executed, but rather a parallel process in which both actions are prepared simultaneously (Cisek 2006; Cisek and Kalaska 2005). These studies suggest that the final movement is the outcome of a competition between the motor plans. This idea has been motivated by evidence suggesting parallel processing during action preparation (Cisek 2006; Cisek and Kalaska 2005; Kornblum et al. 1990; Prescott et al. 1999) and formalized in the affordance competition hypothesis (Cisek 2007; Cisek and Kalaska 2010).

Studies with humans have also indicated that we are capable of simultaneously planning multiple movements. Behavioral evidence comes from studies that examined the effects of noncued targets on movement selection (Gallivan et al. 2015), external perturbations that required rapid switches to an alternative plan (Nashed et al. 2014), and tasks in which participants were forced to move before the target was fully specified (Chapman et al. 2010; Gallivan et al. 2016; Ghez et al. 1997; Haith et al. 2015; Stewart et al. 2014). Neurophysiologically, signatures of parallel planning are evident in studies that showed the modulatory effects of target separation on neural activity (Grent-'t-Jong et al. 2014) as well as cortical inhibition of nonselected movements (Duque et al. 2010; Klein-Flugge and Bestmann 2012). The level at which movement plans compete has been the subject of considerable discussion. Hypotheses range from competition at the level of motor goals (Haith et al. 2015; Wong et al. 2014) to competition at the level of biomechanically specified movements (Cos et al. 2014; Gallivan et al. 2015) and movement control policies (Gallivan et al. 2016).

Simultaneous preparation of multiple movements has been suggested to enable the motor system to respond fast and flexibly to environmental changes (Gallivan et al. 2016; Klapetek et al. 2016; Nashed et al. 2014). However, response selection studies have shown that if the number of response options increases, the reaction time also increases (Hick 1952; Rosenbaum 1980). This suggests that initiating one prepared movement takes less time than initiating one of two prepared movements. When two movements need to be performed simultaneously, response time increases can mainly be ascribed to indirect cueing of movements and, to a lesser extent, to spatial incompatibility of the responses (Diedrichsen et al. 2001; Heuer and Klein 2006; Oliveira and Ivry 2008). Thus, the preparation and execution of multiple movements simultaneously is associated with response time-related costs.

Here, we asked if there are also precision costs related to the simultaneous preparation of multiple movements. A central tenet of cognitive psychology is that the brain is limited in its capacity for performing multiple tasks simultaneously (Moray 1967), with an architecture that allows the system to prioritize among these tasks. These constraints are encapsulated in the concept of a shared central resource. Early theoretical and quantitative models formalized this concept into a relation between the amount of resource dedicated to a goal and the chance of a correct response (Kahneman 1973; Norman and Shallice 1986; Shaw 1978). More recently, work on visual working memory has highlighted the idea that resource sharing has consequences for the precision with which information is internally represented (Bays and Husain 2008; Ma et al. 2014; Palmer 1990; van den Berg et al. 2012; Zhang and Luck 2008). Formally, these models describe the variability with which visual items are represented in memory by a power law: as the number of items increases, the variability associated with the representation of each item increases.

By analogy, if the internal representations of planned movements share a common resource, planning variability should increase as the number of simultaneously planned movements increases. In speeded movements, this increase in planning noise may be observable as an increase in movement variability early in the trajectory (recognizing that feedforward or feedback control processes may influence latter aspects of the trajectory). We tested this prediction by comparing movement variability in conditions requiring one or two movement plans in two different contexts: *1*) within a hand (like moving with one hand to pick up the milk carton on the left or the milk carton on the right) and *2*) between different hands (like using the left or right hand to pick up a single carton of milk). We found that movement plans at both levels of competition were more variable if two movements were prepared at the same time than if only one movement was prepared, consistent with the concept of a limited resource for movement planning.

## METHODS

### Participants and Apparatus

In total, 64 volunteers (21 men and 43 women) aged 19–36 yr (mean: 23.9 yr) participated in one of three experiments (16 volunteers in *experiment 1*, 16 volunteers in *experiment 2*, and 32 volunteers in *experiment 3*). The sample size for all experiments was fixed before testing. All participants were naïve to the research question, had normal hearing ability, and had normal or corrected-to-normal visual acuity. Five participants were left handed; the other 59 participants were right handed. This study was approved by the University College London Research Ethics Committee.

Participants were seated with their chin on a rest and their index finger(s) touched a surface slanted 30° from the horizontal plane. Via a mirror, stimuli displayed on a 21-in. CRT monitor (screen refresh rate: 130 Hz) were reflected into the same plane as the movement surface, although participants could not see their hands. Index finger movement was tracked at 133.3 Hz with an electromagnetic tracking device (3D Guidance trakSTAR, Ascension Technology, Burlington, VT).

### Experiments

#### Experiment 1.

**Fig. 1.**
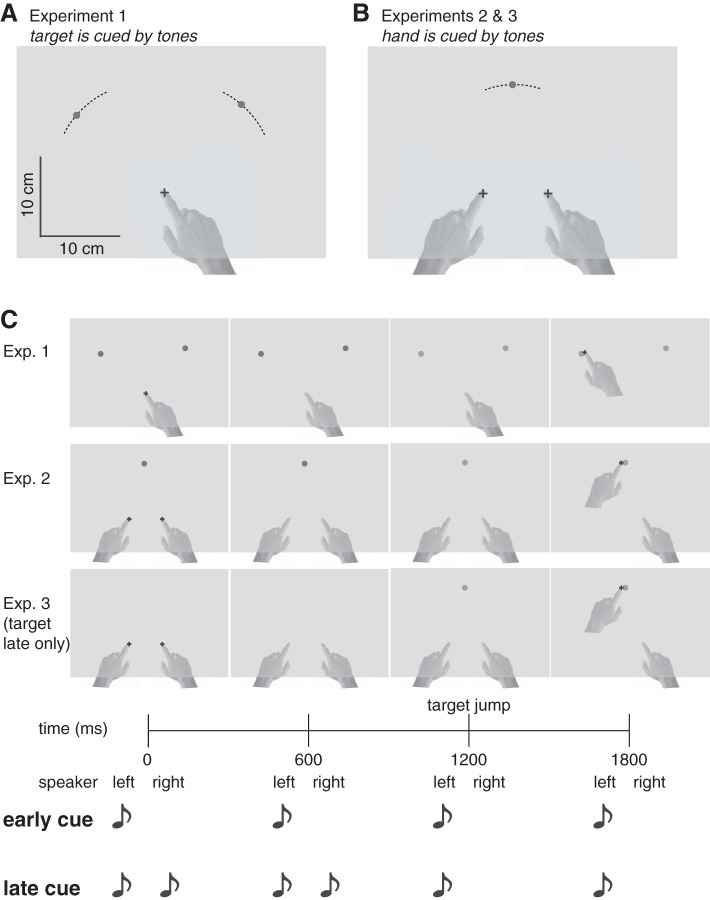
Experimental configurations. *A* and *B*: start point(s) and possible target locations (30° arcs) in *experiment 1* (*A*), in which auditory cues indicated which of two targets to reach to, and *experiments 2* and *3* (*B*), in which cues indicated the response hand. *C*: timing for the two cueing conditions. In these examples, the cue indicates a movement to the left target or with the left hand, respectively. In the early cue condition, this information is provided at the time of the first tone (and coincides with the target onset in *experiments 1* and *2* and the target presented early condition in *experiment 3*). In the late cue condition, this information is only provided at the onset of the imperative. In the target presented late condition in *experiment 3*, the target was presented at the same time as the imperative.

Participants triggered the start of each trial by placing the index finger of their preferred hand on a starting point (crosshair) with the aid of visual feedback (a black circle presented at the fingertip location, 0.4 cm in diameter; Fig. 1*A*). After stable finger placement, visual feedback was removed, and four tones were played at 600-ms intervals over speakers (Creative Inspire T10) placed to the left and right of the setup. Together with the first tone, two potential movement targets were presented. The left target location was randomly drawn from a 30° arc with a 13.33-cm radius from the starting point. The center of the arc was 45° counterclockwise with respect to the midsagittal plane. The right target was always located 90° in the clockwise direction around the starting point with respect to the left target. Targets were red circles, 1 cm in diameter, both of which turned green at the same time as the third tone. To verify if movements were prepared in advance of the third tone, in 20% of the trials the targets changed position (“jumped”) at the same time as the third tone. The new target locations were randomly drawn from the 30° target arc and were at least 10° away from the original locations. Both targets changed by the same distance in the same direction (clockwise or counterclockwise) so that the targets were always 90° apart.

In *experiment 1*, the laterality of the tones (left or right) specified which of the two targets was the movement goal. In the early cue block (50% of trials), all tones were played from a single speaker, thus specifying the goal early in the trial. In the late cue block (50% of trials), the first two tones were played from both speakers and only the last two tones were played from either the left or right speaker (Fig. 1*C*, Table 1). In the late cue condition, left and right targets were randomly interleaved. In the early cue condition, trials to the left and right target were blocked, and the blocks were counterbalanced across participants. Early cued trials were blocked to maximize the chance that participants would plan a single movement. Participants were instructed not to move before the third tone (the imperative) and to hit the specified target by the time of the fourth tone.

The first 10 trials for each cue condition were practice trials during which visual feedback of the index finger position was provided throughout the movement. For the test trials, participants received feedback about the end position of their finger at the end of each response as well as information concerning whether they were successful on that trial. Success required an end point landing inside the target area within 200 ms of the fourth tone. If the movement was initiated before the third tone, the trial was aborted and repeated with new parameters (1.7% of trials repeated). Each participant completed 450 trials in total.

#### Experiment 2.

In *experiment 2*, the tones cued which hand participants had to move toward a single target. There were two starting positions (one for each hand), located 8 cm apart, and a single target, with the location randomly drawn from a 30° arc with a 12.75-cm radius centered halfway between the two starting points (Fig. 1*B*). The laterality of the tones (left or right) specified which of the two hands to move to the target, with early and late cued trials tested in separate blocks. Trials were aborted and repeated with new parameters if the movement was initiated before the third tone or if the wrong hand moved (7.2% of trials repeated). All other features of the design were the same as those in *experiment 1*.

#### Experiment 3.

To disentangle the influences of hand selection and movement planning on movement variability, we incorporated uncertainty about the target location in *experiment 3*. We varied the timing of target presentation relative to cue information. The experimental configuration was the same as that in *experiment 2*, but there were no target jumps. In half the trials, the target was displayed at the first tone (target early); in the other half of the trials, the target was displayed at the third tone (target late). Target early and target late trials were blocked and counterbalanced between participants. For 16 participants, the hand was always cued early (hand early; *experiment 3A*); for 16 other participants, the hand was always cued late (hand late; *experiment 3B*). Again, trials were aborted and repeated with new parameters if the movement was initiated before the third tone or if the wrong hand moved (3.3% of trials repeated). Each participant completed 300 trials in total.

### Analysis

Fingertip position time series were filtered with a low-pass second-order bidirectional Butterworth filter at 10 Hz. Movement speed was obtained by differentiating these series in the two-dimensional movement plane. Movement initiation was defined as the last sample before peak speed in which the speed was <2 cm/s, movement end was defined as the first sample after peak speed in which the speed was <2 cm/s, and movement time was defined as the time between movement initiation and movement end. Response time was defined as the time between the third tone and movement initiation. Movement direction was measured as the angle of the fingertip position with respect to the starting position at the time of peak speed (Churchland et al. 2006a; Messier and Kalaska 1999). On average, peak speed occurred at 44% (SE: 0.4%) of the total movement duration (175 ms, SE: 4 ms after movement initiation). Direction error was defined as the deviation of movement direction from a straight line toward the target. Movement bias was operationalized as the mean direction error and movement variability as the SD of the direction error.

Practice trials, trials for which there was an error with movement registration, and trials for which offline analysis showed that the online registration had failed to detect movement of the wrong hand or toward the wrong target or failed to detect that the initial fingertip position was outside the start position were rejected from analysis (<7% of all trials). The data for one participant in *experiment 1* was discarded as he initially moved in a direction in between the two targets on a large number of trials (behavior not seen in other participants).

Movement variability, movement bias, response time, and movement time across items and conditions were examined with ANOVA and post hoc *t*-tests. We applied a Welch degree of freedom modification for samples of unequal variance. To assess if response time and peak velocity influenced movement variability, we calculated the SD of the initial movement directions for each quartile of the data based on the slowest to fastest response time or peak velocity for each condition and each participant. Linear regression slopes were fit to the distributions of early and late cued no-jump trials for each participant, and we determined if the regression coefficients were different from zero with a one-sample *t*-test. The same procedure was used with response time and absolute movement bias to analyze if they were correlated.

## RESULTS

To examine the influence of the number of movement plans on movement variability, we asked participants to generate rapid reaching movements to one of two visual targets (Fig. 1*A*). In the early cue block, target information was presented well in advance of movement initiation (Fig. 1*C*), allowing participants to prepare a single movement. In the late cue block, this information was only presented at the time movement initiation was signaled; as such, participants should be motivated to prepare movements to both targets given the temporal requirements of the task.

**Fig. 2.**
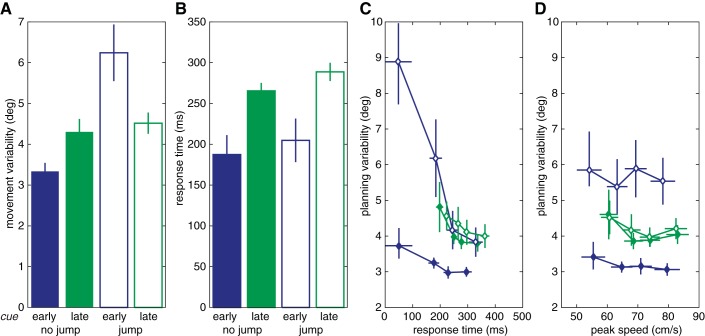
Experiment 1: effect of number of movement plans (within hand). *A* and *B*: movement variability (*A*) and response times (*B*) for target cued early or late. *C* and *D*: movement variability as a function of response time (*C*) and peak speed (*D*) for the different conditions. Error bars indicate ±1 SE.

To confirm that movements were prepared in advance of the imperative, the target unpredictably changed location (jumped) at the time of the imperative on 20% of the trials. If participants were waiting until the imperative to plan their movement, this manipulation should not lead to an increase in movement variability. A target (left or right) × cue (early or late) × jump (no jump or jump) repeated-measures ANOVA showed a main effect for jump, indicating that the initial direction of movement was more variable if the target changed position than if it did not [*F*(1,14) = 20.08, *P* < 0.001]. There was also a significant interaction between cue and jump [*F*(1,14) = 21.56, *P* < 0.001], revealing that the increase in movement variability due to the target jump was actually greater for the early cue than for the late cue (Fig. 2*A*, open bars). Pairwise comparisons showed that target jumps increased movement variability in both the early [*t*(29) = 6.02, *P* < 0.001] and late [*t*(29) = 2.65, *P* = 0.013] cue conditions. The increase in movement variability for the target jump condition confirmed that the participants prepared movements in advance of the imperative.

The key result of this experiment is shown in Fig. 2*A* (solid bars): initial movement direction in the early cue condition was significantly less variable than in the late cue condition [*t*(14) = 4.31, *P* < 0.001; no-jump trials only]. A target (left or right) × target in previous trial (same side or other side) repeated-measures ANOVA showed no significant difference in movement variability within the late cue block between the no-jump trials that switched sides (mean: 4.0°) and when the target was on the same side [mean: 4.4°, *F*(1,14) = 2.10, *P* = 0.169], indicating that it is unlikely that the difference between the early and late cue condition is due to the blocked design. Thus, we can conclude that preparing two movements at the same time results in more variable movements than preparing one movement, suggesting a precision cost related to the concurrent planning of multiple movements that could be explained by movement plans sharing a common resource.

**Fig. 3.**
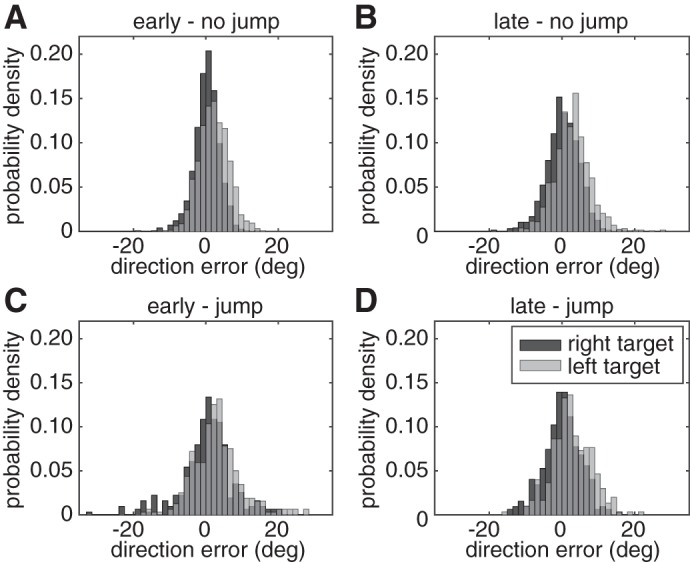
Probability density distributions for direction error in *experiment 1* for early cue no-jump (*A*), late cue no-jump (*B*), early cue jump (*C*), and late cue jump (*D*) conditions. Direction errors to the right target are in dark gray and to the left target in light gray.

The concurrent preparation of multiple movements did not affect movement bias. The results in Fig. 3 show the probability density distributions of direction errors at the time of peak speed. A target (left or right) × cue (early or late) × jump (no jump or jump) repeated-measures ANOVA showed a main effect for target, indicating that movements toward the left target (mean: 2.7°) were 2.5° more to the right than movements to the right target [mean: 0.2°, *F*(1,14) = 8.74, *P* = 0.010]. Thus, participants were aiming slightly less eccentric than straight to the target. Note that if participants were aiming in between the two targets, their initial direction would be 45 and −45° for the left and right target respectively, which was never observed (Fig. 3). There were no other main effects and no interaction effects (all *P* > 0.166), indicating that the movements were similar in the early and late cue conditions. Movement bias was not correlated with response time. The regression coefficients for the relation between response time and movement bias were not significantly different from zero for either early or late cued no-jump trials [early: mean = 6.0 × 10^−7^, *t*(14) = 0.26, *P* = 0.797; and late: mean = −5.1 × 10^−6^, *t*(14) = −1.86, *P* = 0.085].

Differences between the cue conditions in movement variability were accompanied by differences in response time. A target (left or right) × cue (early or late) × jump (no jump or jump) repeated-measures ANOVA showed main effects for cue and jump (Fig. 2*B*). Movements were initiated longer after the imperative if the target was cued late than if it was cued early [*F*(1,14) = 22.86, *P* < 0.001]. As shown in Fig. 2*C*, response time was uncorrelated with movement variability: regression coefficients for the relation between reaction time and movement variability within the early and late cued no-jump trials were not significantly different from zero [early: mean = −2.7 × 10^−5^, *t*(14) = 2.07, *P* = 0.057; and late: mean = −4.4 × 10^−5^, *t*(14) = 1.60, *P* = 0.131]. Thus, response variability was larger in the late cue condition than in the early cue condition even when response times were equated. The same was true for peak speed: the regression coefficients for the relation between peak speed and movement variability were not significantly different from zero for either early or late cued no-jump trials [early: mean = −8.5 × 10^−5^, *t*(14) = 0.58, *P* = 0.573; and late: mean = −1.0 × 10^−4^, *t*(14) = 0.75, *P* = 0.466].

The main effect of jump reflects the fact that response times were longer if the target changed position than if it did not [*F*(1,14) = 14.39, *P* = 0.002]. Together with the quick response times in the no-jump trials (205–270 ms), this provides further evidence that participants planned their movements in advance of the imperative. Moreover, in the early cue jump trials, reaction time was negatively correlated with movement variability [regression coefficient mean: −3.9 × 10^−4^, *t*(14) = 5.65, *P* < 0.001]. A target jump in the early cue condition resulted in almost twice as much initial movement variability as no jump, most likely the result of movements with very short response times being initiated in the direction of the target before it jumped. As response times in the late cue condition were longer, the increase in variability is also smaller. This suggests that although movement initiation was slightly delayed in response to a target jump, it was not delayed until the movement plan was completely updated toward the new target position (Haith et al. 2015). This interpretation is in line with the idea that these two processes operate, at least to some extent, independently (Oostwoud Wijdenes et al. 2011).

### Competing Plans Between Hands

The results of *experiment 1* suggest that movement plans share a common resource. However, two visual targets presented at the same time will also compete for representational resources in the brain (Kastner and Ungerleider 2000). Before a reach, visuospatial attention is directed to the reach target(s) (e.g., Baldauf and Deubel 2010). It is possible that the increase in variability that we observed in *experiment 1* when the target was uncertain is due to noisier representations of the target locations (i.e., divided visuospatial attention). This would not be observed if there was no target uncertainty. In contrast, if the observed variability is related to competing movement plans, variability should also increase when a single target representation is associated with two movement plans, i.e., when the movement may be performed with either the left or right hand (Oliveira et al. 2010). In *experiment 2*, participants were presented with a single target and, in separate blocks, were either provided with an early cue or a late cue specifying the response hand (Fig. 1*B*).

**Fig. 4.**
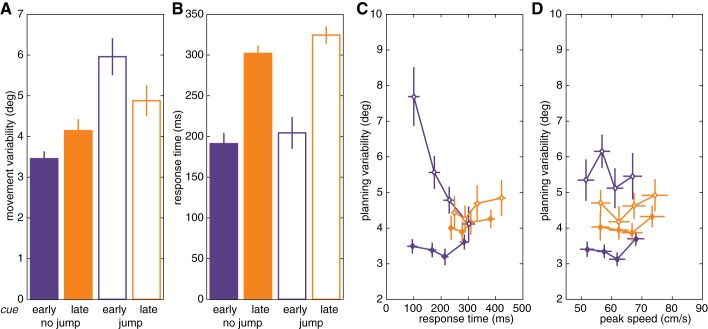
*Experiment 2:* effect of number of movement plans between hands. *A* and *B*: movement variability (*A*) and response times (*B*) for hand cued early or late. *C* and *D*: movement variability as a function of response time (*C*) and peak speed (*D*) for the different conditions.

The results showed that planning movements concurrently with two different hands (*experiments 2*) has very similar consequences to planning two movements with one hand (*experiment 1*). The initial direction of responses was more variable in the late cue condition than in the early cue condition for the no-jump trials [*t*(15) = 3.86, *P* = 0.002; Fig. 4*A*, solid bars]. Thus, we found the same precision-related cost of planning two movements at the same time.

As in *experiment 1*, the jump trials confirmed that the participants planned their responses in advance of the imperative. A hand (left or right) × cue (early or late) × jump (no jump or jump) repeated-measures ANOVA showed that the initial movement direction was more variable for jump trials than for no-jump trials [*F*(1,15) = 43.45, *P* < 0.001]. The interaction between cue and jump was also significant, indicating that the effect of the target jump was again larger for early than late cue trials [*F*(1,15) = 19.91, *P* < 0.001]. Post hoc comparisons showed that jump trials were more variable than no-jump trials for early cued [*t*(31) = 7.34, *P* < 0.001] and late cued [*t*(31) = 4.21, *P* < 0.001] trials. Thus, movements were planned before the imperative, and movement execution was more precise for one planned movement than for two movements planned at the same time.

**Fig. 5.**
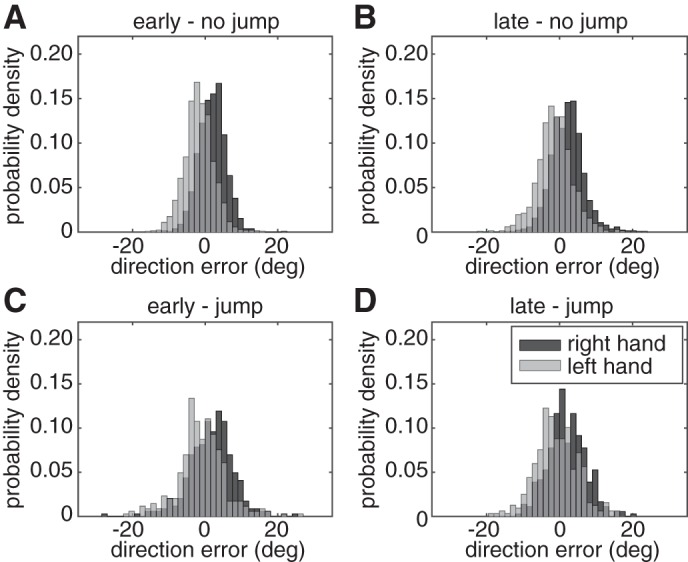
Probability density distributions for direction error in *experiment 2* for early cue no-jump (*A*), late cue no-jump (*B*), early cue jump (*C*) and late cue jump (*D*) conditions. Direction errors with the right hand are in dark gray and with the left hand in light gray.

The movement bias results were also replicated in *experiment 2* (Fig. 5). A hand (left or right) × cue (early or late) × jump (no jump or jump) repeated-measures ANOVA showed a main effect for hand. Movements with the right hand (mean: 1.93°) were more biased toward the right and movements with the left hand were biased to the left [mean: −1.14°, *F*(1,15) = 40.08, *P* < 0.001]. The interaction effect between hand and jump was close to significant [*F*(1,14) = 3.79, *P* = 0.071], indicating that the bias for both hands was closer to zero for jump trials than for no-jump trials. The other effects were not significant (all *P* > 0.189). The regression coefficients for the relation between response time and movement bias were also not significantly different from zero for either early or late cued no-jump trials [early: mean = 1.1 × 10^−6^, *t*(15) = 0.69, *P* = 0.501; and late: mean = 3.1 × 10^−7^, *t*(15) = 0.11, *P* = 0.915].

Responses were initiated faster in the early cue condition than in the late cue condition and also faster if the target did not jump than if it jumped [*F*(1,15) = 102.9, *P* < 0.001, and *F*(1,15) = 13.47, *P* = 0.002, respectively; see also Fig. 4*B*]. Response times were shorter and peak speeds were lower in the early cue condition than in the late cue condition. There was no correlation between timing parameters and movement variability in the no-jump conditions {regression coefficients for response time [early: mean = −6.0 × 10^−6^, *t*(15) = 0.36, *P* = 0.726; and late: mean = 3.7 × 10^−5^; *t*(15) = 1.32, *P* = 0.206] and peak speed [early: mean = 6.4 × 10^−5^; *t*(15) = 0.36, *P* = 0.722; and late: mean = 2.3 × 10^−4^, *t*(15) = 1.20, *P* = 0.249] were not significantly different from zero}. Thus, as in *experiment 1*, the target jump resulted in delayed movement initiation and higher movement variability.

### General Preparedness

Our central hypothesis was that the increased variability in two-plan conditions is due to competition between motor plans. We assume that these plans entail some specification of desired trajectories, either in egocentric or joint space (Gallivan et al. 2015; Shadmehr and Mussa-Ivaldi 1994). An alternative possibility is that the differences in variability reflect a more general preparedness of the hand to move that doesn't require trajectory specification, such as hand selection. To examine if hand preparedness influences movement variability, we conducted *experiment 3*, in which we manipulated the timing of the target presentation while the hand was cued early (*experiment 3A*) or late (*experiment 3B*). Given that a trajectory cannot be specified until the target is known, we predicted that the increase in variability associated with multiple plans would only be observed when the target was presented in advance of the imperative signal (early) and would not be found when the target was presented at the time of the imperative signal (late).

**Fig. 6.**
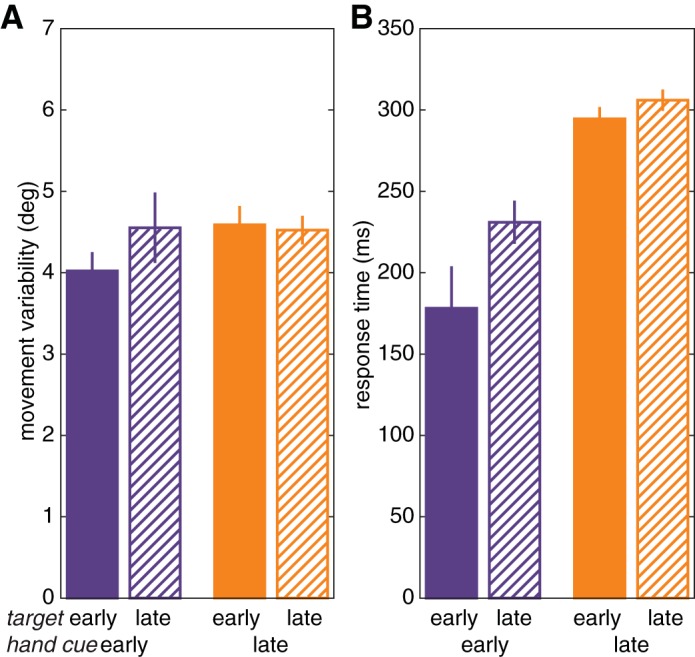
*Experiment 3*: effect of general preparedness. *A* and *B*: movement variability (*A*) and response time (*B*) for *experiment 3A*, in which there was no hand uncertainty because the hand was cued early (purple), and *experiment 3B*, in which movements with both hands had to be prepared because the hand was cued late (orange). Solid bars indicate conditions in which there was no target uncertainty because the target was presented at the first tone (early), as in the no-jump condition of *experiment 2*. Hatched bars indicate conditions in which the target was presented at the time of the imperative (late).

When the target was presented early, movements were more variable if the hand was cued late than if the hand was cued early, replicating the findings of *experiment 2* (between participants, Welch two-sample *t*-test: *t* = 2.21, *P* = 0.031; Fig. 6*A*, solid bars). The critical test in this experiment involves the conditions in which the target was presented at the time of the imperative (late), given our assumption that this would preclude movement planning. Here, there was no effect of hand cue timing: variability was similar for the hand early and hand late conditions when the target was presented late (Welch two-sample *t*-test: *t* = 0.06, *P* = 0.950; Fig. 6*A*, hatched bars). Moreover, variability in these conditions was greater than in the target early/hand early condition [target late/hand early, paired-samples *t*-test: *t*(31) = 2.36, *P* = 0.025; target late/hand late, Welch two sample *t*-test: *t* = 2.15, *P* = 0.036], suggesting that movement variability is reduced when the forthcoming movement can be completely specified.

Although this was not a straightforward reaction time task, the response time data were congruent with what one would expect based on results of speeded response paradigms (Rosenbaum 1980). Responses were initiated earlier if the target was presented early than if it was presented late, an effect observed for both hand cued early [*t*(31) = 3.44, *P* = 0.002] and hand cued late [*t*(31) = 2.21, *P* = 0.034; Fig. 6*B*]. Response times were also faster if the hand was cued early than if it was cued late [target early: *t*(36.40) = 5.91, *P* < 0.001; and target late: *t*(46.33) = 6.97, *P* < 0.001]. Thus, in line with previous findings, responses were faster when more information was specified before the imperative, whereas late specification of the hand delayed responses more than late specification of target position.

## DISCUSSION

We examined if movement plans share a common resource by testing if planning variability was affected by the number of movements prepared concurrently. We compared initial movement variability in an early cue condition, where preparation should be limited to a single movement, to a late cue condition, in which we assumed participants would have to prepare two different movements, given the constraints on reaction time. As predicted, the initial movement direction in the early cue condition was less variable than in the late cue condition, regardless of whether the two plans corresponded to two different movements of the same hand (*experiment 1*; Fig. 2) or movements with the two different hands (*experiment 2*; Fig. 4).

To confirm that participants' engaged in advance planning, we included control conditions in which the target changed position at the time of the imperative. If the process of planning a movement in all conditions began only at the imperative, the previous location of the target would be irrelevant and there would be no cost in the target jump conditions. In contrast to this prediction, the initial movement variability and response time increased in the target jump conditions (Figs. 2*A* and 3*A*, open bars). Thus, it appears that, consistent with our assumption, movement preparation began before the imperative. The increase in variability and response time observed when the target jumped is likely due to the need to update plans. For very short response times, movements may be initially directed toward the old target location or to locations between the old and the new target (Haith et al. 2015; Van Sonderen and Denier van der Gon 1991). The increase in variability was larger in the early cue condition because here movements were initiated earlier on average and, therefore, when target location was more uncertain.

Our results demonstrate a precision cost related to simultaneously preparing multiple movements. This precision cost resembles the cost observed in studies of visual working memory (Bays 2015; Ma et al. 2014), although the increase in variability observed in the present study is somewhat smaller than typically observed in studies of visual working memory. In working memory studies, variability in recall has been found to increase steadily and in a continuous manner as the number of items in memory increases (Bays and Husain 2008; Bays et al. 2009; Palmer 1990; van den Berg et al. 2012; Wilken and Ma 2004). Moreover, salient or goal-relevant items are stored with increased precision but at a cost to the memorability of other items in memory (Bays and Husain 2008; Bays et al. 2011; Gorgoraptis et al. 2011). These results are consistent with the hypothesis that working memory is constrained by a limited resource that is shared between the representations.

Our results point to a similar constraint for multiple competing movement plans when there are multiple task-relevant targets (Chapman et al. 2010; Cisek 2006; Duque et al. 2010; Gallivan et al. 2015) or when movements to a single target can be accomplished with either hand (Oliveira et al. 2010). We suggest that the neural mechanisms that cause the representational noise to increase for multiple items in working memory also impacts the fidelity of the representation of multiple movement plans. That is, we propose that neurons face similar energetic constraints when representing multiple visual features or movements and that the way in which this problem is resolved is similar for the two systems. The variability increase for two competing movement plans may be less than for two competing items in working memory because for reaching, resources can be dynamically reallocated after the late cue. It is also possible that there is a different power law constant for movement planning resources than for working memory resources. Additionally, other factors than the amount of resources dedicated to the movement plan, for which the power law relationship does not hold, might affect movement variability, for example, online movement corrections.

A neural model in which feature representations are based on population coding, with the limited shared resource construct implemented by normalization of population activity, can reproduce the specific distributions of errors observed in visual working memory (Bays 2014). As the number of items increases, the firing rates associated with each item decrease, and it becomes harder to distinguish spikes representing individual items from noise; thus, working memory variability increases. This model is supported by neurophysiological findings showing that the firing rates correlated with features of remembered items decreases as the number of items increases (Buschman et al. 2011). In addition, in human functional MRI, the amplitude of the BOLD response associated with target representations in working memory decreases as the number of represented items increases from one to two (Sprague et al. 2014). Mirroring the findings on firing rates for items in working memory, nonhuman primate electrophysiology studies have shown that average firing rates decrease and firing rate variability increases when the number of response alternatives increases (Churchland and Ditterich 2012; Churchland et al. 2011). This is consistent with our hypothesis that the same neural constraints are responsible for the increase in planning noise that we observed when multiple plans are prepared simultaneously.

We hypothesize that competition for resources among movement plans takes place in areas associated with movement preparation, such as the premotor cortex and posterior parietal cortex (Cui and Andersen 2007; Roland et al. 1980; Snyder et al. 1997). Areas in the intraparietal sulcus and premotor cortex have been shown to represent actions independent of the effector that will execute the movement (Gallivan et al. 2013; Medendorp 2004), making them possible loci for competition between motor plans. Top-down allocation of resources could reflect processing in a domain-general area such as the prefrontal cortex.

The results of *experiment 3* were especially interesting in showing that there was no difference in variability between early cue and late cue conditions when the target position was unknown before the imperative (Fig. 4). This result suggests that competition does not arise at the level of a general readiness to move. Instead, the precision benefit for planning a single movement appears to be present only when a specific movement can be prepared. However, the exact level of specification remains to be established: resources might be distributed across high-level representations of motor goals (Wong et al. 2014) or low-level fully specified movement trajectories (Cos et al. 2014; Gallivan et al. 2015).

One might wonder why movements were not more variable in the conditions in which the target was presented late compared with when the target was specified early but the hand was specified late. In the former, it was not possible to plan anything before the imperative, whereas in the latter, there were two competing plans. We speculate that this is related to the differences in response times, with movement initiation delayed until variance drops below a criterion value (Churchland et al. 2006b).

Besides uncovering a precision-related cost associated with concurrently planned movements, we also replicated the well-known response time-related cost. Response time was aided by early specification of parameters of the movement. Responses were initiated sooner after the imperative in the early cue condition than in the late cue condition and also sooner in the target presented early condition than in the target presented late condition (Figs. 2*B*, 4*B*, and 6*B*). This is in accordance with seminal studies that have shown that reaction times are faster if there are fewer possible targets and if more information is specified before the imperative (Goodman and Kelso 1980; Hick 1952; Rosenbaum 1980). Identification of the side that the tone was coming from might have additionally affected the response times in the late cue condition. The relationship between the number of cues and reaction time has been formalized in a capacity-sharing model with similarities to a shared resource account (Pellizzer and Hedges 2003).

In previous studies in which the target was specified only after the imperative, it has been reported that the movement initially follows a path that is the average of movement paths toward all of the possible targets (Chapman et al. 2010; Stewart et al. 2014). In our study, movement averaging could in theory have contributed to the observed variability in *experiment 1*. However, at odds with this hypothesis, movement biases were not affected by cue condition. It is also not consistent with the results of *experiment 2* where the difference between movement plans was based on the number of candidate hands (one or two) for the forthcoming response.

In summary, we propose that the level of planning noise, manifest in the form of movement variability (Afshar and Shenoy 2006; Chaisanguanthum et al. 2014; Churchland et al. 2006a; van Beers 2009), is a function of the amount of resources dedicated to a motor plan. Similar to working memory, resources may be allocated flexibly, depending on the number of alternative movement plans. Importantly, our results indicate a precision constraint on the ability to prepare multiple movements simultaneously. If too many movements are prepared at the same time, each representation will suffer from high levels of noise. Therefore, preparing a small number of plans at one time may be optimal for generating fast, accurate movements.

**Table 1.** Properties of the experiments

	What Is Cued?	Timing Cue	Timing Target(s)	Target Jumps
*Experiment 1*	Target	Early/late	Early	20%
*Experiment 2*	Hand	Early/late	Early	20%
*Experiment 3A*	Hand	Early	Early/late	No
*Experiment 3B*	Hand	Late	Early/late	No

## GRANTS

This work was supported by the Wellcome Trust and the Van Coeverden Adriani Stichting.

## DISCLOSURES

No conflicts of interest, financial or otherwise, are declared by the author(s).

## AUTHOR CONTRIBUTIONS

L.O.W., R.B.I., and P.M.B. conception and design of research; L.O.W. performed experiments; L.O.W. analyzed data; L.O.W., R.B.I., and P.M.B. interpreted results of experiments; L.O.W. prepared figures; L.O.W. and P.M.B. drafted manuscript; L.O.W., R.B.I., and P.M.B. edited and revised manuscript; L.O.W., R.B.I., and P.M.B. approved final version of manuscript.

## References

[B1] Afshar A, Shenoy KV A central source of movement variability. Neuron 52: 1085–1096, 2006. 17178410 10.1016/j.neuron.2006.10.034 PMC1941679

[B2] Baldauf D, Deubel H Attentional landscapes in reaching and grasping. Vis Res 50: 999–1013, 2010. 20219518 10.1016/j.visres.2010.02.008

[B3] Bays PM Noise in neural populations accounts for errors in working memory. J Neurosci 34: 3632–3645, 2014. 24599462 10.1523/JNEUROSCI.3204-13.2014 PMC3942580

[B4] Bays PM Spikes not slots: noise in neural populations limits working memory. Trends Cogn Sci 19: 431–438, 2015. 26160026 10.1016/j.tics.2015.06.004

[B5] Bays PM, Catalao RFG, Husain M The precision of visual working memory is set by allocation of a shared resource. J Vis 9: 7–7, 2009. 19810788 10.1167/9.10.7 PMC3118422

[B6] Bays PM, Gorgoraptis N, Wee N, Marshall L, Husain M Temporal dynamics of encoding, storage, and reallocation of visual working memory. J Vis 11: 6–6, 2011. 21911739 10.1167/11.10.6 PMC3401684

[B7] Bays PM, Husain M Dynamic shifts of limited working memory resources in human vision. Science 321: 851–854, 2008. 18687968 10.1126/science.1158023 PMC2532743

[B8] Buschman TJ, Siegel M, Roy JE, Miller EK Neural substrates of cognitive capacity limitations. Proc Natl Acad Sci USA 108: 11252–11255, 2011. 21690375 10.1073/pnas.1104666108 PMC3131328

[B9] Chaisanguanthum KS, Shen HH, Sabes PN Motor variability arises from a slow random walk in neural state. J Neurosci 34: 12071–12080, 2014. 25186752 10.1523/JNEUROSCI.3001-13.2014 PMC4152607

[B10] Chapman CS, Gallivan JP, Wood DK, Milne JL, Culham JC, Goodale MA Reaching for the unknown: multiple target encoding and real-time decision-making in a rapid reach task. Cognition 116: 168–176, 2010. 20471007 10.1016/j.cognition.2010.04.008

[B11] Churchland AK, Ditterich J New advances in understanding decisions among multiple alternatives. Curr Opin Neurobiol 22: 920–926, 2012. 22554881 10.1016/j.conb.2012.04.009 PMC3422607

[B12] Churchland AK, Kiani R, Chaudhuri R, Wang XJ, Pouget A, Shadlen MN Variance as a Signature of Neural Computations during Decision Making. Neuron 69: 818–831, 2011. 21338889 10.1016/j.neuron.2010.12.037 PMC3066020

[B13] Churchland MM, Afshar A, Shenoy KV A central source of movement variability. Neuron 52: 1085–1096, 2006a. 17178410 10.1016/j.neuron.2006.10.034 PMC1941679

[B14] Churchland MM, Yu BM, Ryu SI, Santhanam G, Shenoy KV Neural variability in premotor cortex provides a signature of motor preparation. J Neurosci 26: 3697–3712, 2006b. 16597724 10.1523/JNEUROSCI.3762-05.2006 PMC6674116

[B15] Cisek P Integrated neural processes for defining potential actions and deciding between them: a computational model. J Neurosci 26: 9761–9770, 2006. 16988047 10.1523/JNEUROSCI.5605-05.2006 PMC6674435

[B16] Cisek P Cortical mechanisms of action selection: the affordance competition hypothesis. Philos Trans R Soc B Biol Sci 362: 1585–1599, 2007. 10.1098/rstb.2007.2054 PMC2440773 17428779

[B17] Cisek P, Kalaska JF Neural correlates of reaching decisions in dorsal premotor cortex: specification of multiple direction choices and final selection of action. Neuron 45: 801–814, 2005. 15748854 10.1016/j.neuron.2005.01.027

[B18] Cisek P, Kalaska JF Neural mechanisms for interacting with a world full of action choices. Annu Rev Neurosci 33: 269–298, 2010. 20345247 10.1146/annurev.neuro.051508.135409

[B19] Cos I, Duque J, Cisek P Rapid prediction of biomechanical costs during action decisions. J Neurophysiol 112: 1256–1266, 2014. 24899673 10.1152/jn.00147.2014

[B20] Cui H, Andersen RA Posterior parietal cortex encodes autonomously selected motor plans. Neuron 56: 552–559, 2007. 17988637 10.1016/j.neuron.2007.09.031 PMC2651089

[B21] Diedrichsen J, Hazeltine E, Kennerley S, Ivry RB Moving to directly cued locations abolishes spatial interference during bimanual actions. Psychol Sci 12: 493–498, 2001. 11760137 10.1111/1467-9280.00391

[B22] Duque J, Lew D, Mazzocchio R, Olivier E, Ivry RB Evidence for two concurrent inhibitory mechanisms during response preparation. J Neurosci 30: 3793–3802, 2010. 20220014 10.1523/JNEUROSCI.5722-09.2010 PMC2852647

[B23] Gallivan JP, Barton KS, Chapman CS, Wolpert DM, Flanagan JR Action plan co-optimization reveals the parallel encoding of competing reach movements. Nat Commun 6: 7428, 2015. 26130029 10.1038/ncomms8428 PMC4502063

[B24] Gallivan JP, Logan L, Wolpert DM, Flanagan JR Parallel specification of competing sensorimotor control policies for alternative action options. Nat Neurosci 19: 320–326, 2016. 26752159 10.1038/nn.4214 PMC6103436

[B25] Gallivan JP, McLean DA, Flanagan JR, Culham JC Where one hand meets the other: limb-specific and action-dependent movement plans decoded from preparatory signals in single human frontoparietal brain areas. J Neurosci 33: 1991–2008, 2013. 23365237 10.1523/JNEUROSCI.0541-12.2013 PMC6619126

[B26] Ghez C, Favilla M, Ghilardi MF, Gordon J, Bermejo R, Pullman S Discrete and continuous planning of hand movements and isometric force trajectories. Exp Brain Res 115: 217–233, 1997. 9224851 10.1007/pl00005692

[B27] Goodman D, Kelso JA Are movements prepared in parts? Not under compatible (naturalized) conditions. J Exp Psychol Gen 109: 475–495, 1980. 6449532 10.1037//0096-3445.109.4.475

[B28] Gorgoraptis N, Catalao RFG, Bays PM, Husain M Dynamic updating of working memory resources for visual objects. J Neurosci 31: 8502–8511, 2011. 21653854 10.1523/JNEUROSCI.0208-11.2011 PMC3124758

[B29] Grent-'t-Jong T, Oostenveld R, Jensen O, Medendorp WP, Praamstra P Competitive interactions in sensorimotor cortex: oscillations express separation between alternative movement targets. J Neurophysiol 112: 224–232, 2014. 24760786 10.1152/jn.00127.2014 PMC4064405

[B30] Haith AM, Huberdeau DM, Krakauer JW Hedging your bets: intermediate movements as optimal behavior in the context of an incomplete decision. PLos Comput Biol 11: e1004171, 2015. 25821964 10.1371/journal.pcbi.1004171 PMC4379031

[B31] Heuer H, Klein W The influence of movement cues on intermanual interactions. Psychol Res 70: 229–244, 2006. 16082546 10.1007/s00426-005-0218-9

[B32] Hick WE On the rate of gain of information. Q J Exp Psychol 4: 11–26, 1952.

[B33] Kahneman D Attention and Effort. Englewood Cliffs, NJ: Prentice-Hall, 1973.

[B34] Kastner S, Ungerleider LG Mechanisms of visual attention in the human cortex. Annu Rev Neurosci 23: 315–341, 2000. 10845067 10.1146/annurev.neuro.23.1.315

[B35] Klapetek A, Jonikaitis D, Deubel H Attention allocation before antisaccades. J Vis 16: 11, 2016. 26790843 10.1167/16.1.11

[B36] Klein-Flugge MC, Bestmann S Time-dependent changes in human corticospinal excitability reveal value-based competition for action during decision processing. J Neurosci 32: 8373–8382, 2012. 22699917 10.1523/JNEUROSCI.0270-12.2012 PMC3399779

[B37] Kornblum S, Hasbroucq T, Osman A Dimensional overlap: cognitive basis for stimulus-response compatibility–a model and taxonomy. Psychol Rev 97: 253–270, 1990. 2186425 10.1037/0033-295x.97.2.253

[B38] Ma WJ, Husain M, Bays PM Changing concepts of working memory. Nat Neurosci 17: 347–356, 2014. 24569831 10.1038/nn.3655 PMC4159388

[B39] Medendorp WP Integration of target and effector information in human posterior parietal cortex for the planning of action. J Neurophysiol 93: 954–962, 2004. 15356184 10.1152/jn.00725.2004

[B40] Messier J, Kalaska JF Comparison of variability of initial kinematics and endpoints of reaching movements. Exp Brain Res 125: 139–152, 1999. 10204767 10.1007/s002210050669

[B41] Moray N Where is capacity limited? A survey and a model. Acta Psychol (Amst) 27: 84–92, 1967. 6062244 10.1016/0001-6918(67)90048-0

[B42] Nashed JY, Crevecoeur F, Scott SH Rapid online selection between multiple motor plans. J Neurosci 34: 1769–1780, 2014. 24478359 10.1523/JNEUROSCI.3063-13.2014 PMC8186509

[B43] Norman DA, Shallice T Attention to Action: Willed and Automatic Control of Behavior. New York: Springer Science+Business Media, 1986.

[B44] Oliveira FTP, Diedrichsen J, Verstynen T, Duque J, Ivry RB Transcranial magnetic stimulation of posterior parietal cortex affects decisions of hand choice. Proc Natl Acad Sci USA 107: 17751–17756, 2010. 20876098 10.1073/pnas.1006223107 PMC2955129

[B45] Oliveira FTP, Ivry RB The representation of action: insights from bimanual coordination. Curr Dir Psychol Sci 17: 130–135, 2008. 19606276 10.1111/j.1467-8721.2008.00562.x PMC2709871

[B46] Oostwoud Wijdenes L, Brenner E, Smeets JBJ Fast and fine-tuned corrections when the target of a hand movement is displaced. Exp Brain Res 214: 453–462, 2011. 21874536 10.1007/s00221-011-2843-4 PMC3178780

[B47] Palmer J Attentional limits on the perception and memory of visual information. J Exp Psychol Hum Percept Perform 16: 332–350, 1990. 2142203 10.1037//0096-1523.16.2.332

[B48] Pellizzer G, Hedges JH Motor planning: effect of directional uncertainty with discrete spatial cues. Exp Brain Res 150: 276–289, 2003. 12684730 10.1007/s00221-003-1453-1

[B49] Prescott TJ, Redgrave P, Gurney K Layered control architectures in robots and vertebrates. Adapt Behav 7: 99–127, 1999.

[B50] Roland PE, Larsen B, Lassen NA, Skinhøj E Supplementary motor area and other cortical areas in organization of voluntary movements in man. J Neurophysiol 43: 118–136, 1980. 7351547 10.1152/jn.1980.43.1.118

[B51] Rosenbaum DA Human movement initiation: specification of arm, direction, and extent. J Exp Psychol Gen 109: 444–474, 1980. 6449531 10.1037//0096-3445.109.4.444

[B52] Shadmehr R, Mussa-Ivaldi FA Adaptive representation of dynamics during learning of a motor task. J Neurosci 14: 3208–3224, 1994. 8182467 10.1523/JNEUROSCI.14-05-03208.1994 PMC6577492

[B53] Shaw ML A capacity allocation model for reaction time. J Exp Psychol Hum Percept Perform 4: 586–598, 1978.

[B54] Snyder LH, Batista AP, Andersen RA Coding of intention in the posterior parietal cortex. Nature 386: 167–170, 1997. 9062187 10.1038/386167a0

[B55] Song JH, Nakayama K Hidden cognitive states revealed in choice reaching tasks. Trends Cogn Sci 13: 360–366, 2009. 19647475 10.1016/j.tics.2009.04.009

[B56] Sprague TC, Ester EF, Serences JT Reconstructions of information in visual spatial working memory degrade with memory load. Curr Biol 24: 2174–2180, 2014. 25201683 10.1016/j.cub.2014.07.066 PMC4181677

[B57] Stewart BM, Gallivan JP, Baugh LA, Flanagan JR Motor, not visual, encoding of potential reach targets. Curr Biol 24: R953–954, 2014. 25291634 10.1016/j.cub.2014.08.046

[B58] van Beers RJ Motor learning is optimally tuned to the properties of motor noise. Neuron 63: 406–417, 2009. 19679079 10.1016/j.neuron.2009.06.025

[B59] van den Berg R, Shin H, Chou WC, George R, Ma WJ Variability in encoding precision accounts for visual short-term memory limitations. Proc Natl Acad Sci USA 109: 8780–8785, 2012. 22582168 10.1073/pnas.1117465109 PMC3365149

[B60] Van Sonderen JF, Denier van der Gon JJ Reaction-time-dependent differences in the initial movement direction of fast goal-directed arm movements. Hum Movement Sci 10: 713–726, 1991.

[B61] Wilken P, Ma WJ A detection theory account of change detection. J Vis 4: 1120–1135, 2004. 15669916 10.1167/4.12.11

[B62] Wong AL, Haith AM, Krakauer JW Motor planning. Neuroscientist 21: 385–398, 2015. 24981338 10.1177/1073858414541484

[B63] Zhang W, Luck SJ Discrete fixed-resolution representations in visual working memory. Nature 453: 233–235, 2008. 18385672 10.1038/nature06860 PMC2588137

